# Anti-aging mechanism and effect of treatment with raw and wine-steamed *Polygonatum sibiricum* on D-galactose-induced aging in mice by inhibiting oxidative stress and modulating gut microbiota

**DOI:** 10.3389/fphar.2024.1335786

**Published:** 2024-05-07

**Authors:** Ruixue Zhong, Ling Shen, Yilin Fan, Qiaomei Luo, Ran Hong, Xiaoli Sun, Xia Zhou, Jun Wan

**Affiliations:** ^1^ Sichuan Provincial Orthopedic Hospital, Chengdu, China; ^2^ College of Life Science and Engineering, Southwest Jiaotong University, Chengdu, China

**Keywords:** *Polygonatum sibiricum*, oxidative stress, intestinal flora, Kelch-like ECH-associated protein 1, nuclear factor erythroid 2-related factor 2, heme oxygenase 1

## Abstract

**Background:**

*Polygonatum sibiricum* (PS) is a traditional Chinese medicine (TCM) first recorded in *Mingyi Bielu*. The book documents that PS can nourish five internal organs, be taken for a long time, relax the body and prolong lifespan. Presently, PS is widely used in TCM to prevent premature graying of hair. Based on TCM theory and clinical trials, the wine steaming processed product from PS provides a better effect. However, no published study has elucidated the anti-aging mechanism.

**Purpose:**

The study aim was to investigate the anti-aging mechanism of PS and its wine steaming processed product in mice, specifically focusing on the effect of D-galactose (D-gal) surrounding the intestinal flora and the Kelch-like ECH-associated protein 1-nuclear factor erythroid 2-related factor 2-antioxidant response elements (Keap1/Nrf2/ARE) pathway.

**Methods:**

The chemical components in Raw PS (RPS) and Wine-steamed PS (WPS) were identified by ultra-performance liquid chromatography–hybrid quadrupole-Orbitrap high-resolution mass spectrometry (UPLC-Q-Orbitrap HRMS). An aging model using Kunming mice was established through intraperitoneally injected D-gal. Concentrations of RPS and WPS at 5, 10, or 15 g/kg/day levels were administered intragastrically, respectively. The body weight, liver and spleen indexes, superoxide dismutase (SOD), glutathione peroxidase (GSH-PX), and malondialdehyde (MDA) activities in serum and brain tissue were recorded. Hematoxylin and eosin (HE) stained brain tissue was histopathologically examined. The expressions of Keap1, Nrf2 and heme oxygenase 1 (HO-1) in the brain tissue at the mRNA and protein levels were respectively detected by reverse transcription-polymerase chain reaction (RT-PCR) and western blot (WB). Moreover, an Illumina Hiseq platform was used for 16S ribosomal RNA (16S rRNA) high-throughput sequencing to evaluate the proportions of intestinal flora in aging mice.

**Results:**

The proportions of saccharides, flavonoids, and triterpene acids were different between RPS and WPS. In the aging model mice, WPS outperformed RPS in improving body weight and mental state by increasing the spleen index, SOD and GSH-PX activities, decreasing the liver index and MDA activities, and restoring the histopathological morphology in D-gal-induced aging mice. At the mRNA levels, RPS and WPS significantly reduced the expression of Keap1 and increased the expressions of Nrf2 and HO-1. The trend in protein expressions was similar to that of the mRNA results, and WPS had a stronger effect than RPS. Fecal microbiota analysis showed that RPS and WPS restored intestinal microbiota proportions to normal levels.

**Conclusion:**

The results demonstrated that PS and its WPS had a positive effect in relieving oxidative stress in aging mice. WPS outperformed RPS, which might be related to the activation of the Keap1/Nrf2/ARE pathway and regulation of intestinal flora.

## Graphical Abstract

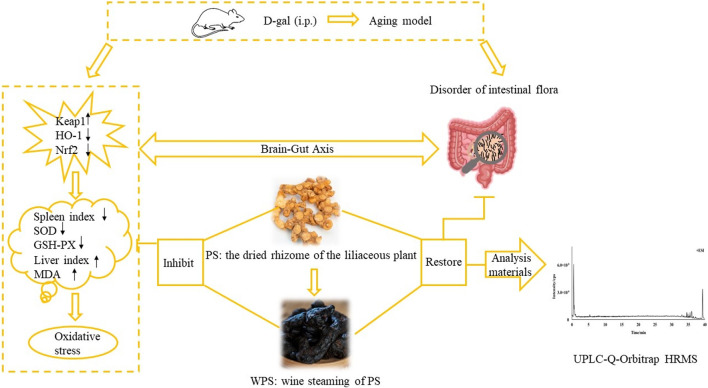

## 1 Introduction

*Polygonatum sibiricum* (PS) is the dried rhizome of the liliaceous plant. There are three species of *Polygonati Rhizoma* widely used in traditional Chinese herbal medicines: *P*. *kingianum* Coll. et Hemsl., *P*. *sibiricum* Red., *P. cyrtonema* Hua., known as “Huangjing” (Xiaowei et al., 2019; Chinese Pharmacopoeia Commission, 2020). PS has the effect of boosting Qi and nourishing Yin, strengthening the spleen, moistening the lung, and benefiting the kidney. In TCM, PS was first recorded by *Mingyi Bielu*, and was listed to the “Top Grade” herb in *Shennong Bencao Jing* (Zhao et al., 2018; Han et al., 2020). As a “medicine food homology” plant, many bioactive ingredients have been confirmed in PS, including polysaccharides, steroidal saponins, amino acids, flavonoids, anthraquinones, and alkaloids (Zeng et al., 2020). Modern pharmacological studies have demonstrated that PS decreases blood glucose (Ma et al., 2022) and blood pressure (Su et al., 2022), prevents atherosclerosis (Zhu et al., 2015), enhances immunity (Sun et al., 2022), and has anti-fatigue (Shen et al., 2021), anti-tumor (Li et al., 2022), and anti-aging effects (Chen et al., 2020). According to the previous study, the polysaccharide of PS could improve D-gal-induced aging rats’ state by up-regulating the expression of Klotho, and down-regulating the expression of FOXO3a in the mRNA and protein levels (Zheng, 2020). Besides, *in vitro*, 10% drug-containing serum of *Polygonati Rhizoma* could significantly decrease the reactive oxygen species (ROS) level. At the mRNA and protein levels, ataxia-telangiectasia mutated gene (ATM), ATM and Rad3-related kinase, check point 1, and check point 2 were significantly downregulated to intervene endothelial progenitor cells aging process (Qin et al., 2019). Wang et al. (2008) found that PS can effectively increase the activities of SOD, GSH-PX, Na^+^-K^+^-ATP, and Ca^2+^-ATP, and reduce the content of MDA in the brain tissue of aging mice.

However, Raw *Polygonatum sibiricum* (RPS) has a hemp taste, and when taken orally, the mouth and tongue are numbed and the throat is stimulated. Usually, PS needs to be processed before use in the clinic (Ren et al., 2020). Processing is common in TCM and has a crucial role in improving clinical efficacy and adaptation. Processing methods of PS include “nine steaming and nine drying,” “steaming,” and “wine steaming” (Yang and Gong, 2017). The Chinese Pharmacopeia (2020 edition) documents the “wine steaming” processing method, which involves taking 100 g of RPS, adding 20 g of wine, cutting thick slices, and drying (Chinese Pharmacopoeia Commission, 2020). Wine-steamed *Polygonatum sibiricum* (WPS) was obtained. The irritation and side effects of RPS are eliminated, and the drug function is changed after processing. The wining process accelerates the accumulation of effective ingredients and elimination of mucus (Lin et al., 2021). Nevertheless, at present, most studies of PS processing focus on the chemical composition change before and after processing, and the specific chemicals responsible for the therapeutic effect and scientific explanation of how the processing alters the therapeutic effect are not clear, which limits development of PS products.

As an inevitable physiological process, aging can lead to the gradual loss of some bodily functions, among which cognitive decline has become one of the biggest health threats to the elderly. In recent years, there have been many studies on the mechanism of aging, such as the telomere theory, free-radical theory, and metabolic imbalance theory (Rehman et al., 2017). Moreover, the free-radical theory shows that serious lesions can occur when excessive free-radicals are produced or the scavenging ability is increased from the human body (Liochev, 2013). Therefore, scavenging of excessive ROS free radicals has important physiological significance for slowing aging. There are two ways for organisms to remove excessive ROS free radicals: the antioxidant enzyme system and the non-enzymatic antioxidant system. When ROS is overproduced, the body’s antioxidant enzyme system is first activated (Sugino, 2006). SOD, GSH-PX, MDA and other enzymes have an important role in the body’s defensive mechanisms (Atmaca et al., 2008).

Studies have shown that the Keap1/Nrf2/ARE pathway is an essential signaling pathway and one of the vital mechanisms of antioxidants when the body is stimulated by oxidation (Chakkittukandiyil et al., 2022). In the normal state of the human body, Nrf2 binds to Keap1 in the cytoplasm, and Nrf2 protein is degraded so that a low level of Nrf2 is maintained. Under stressful conditions, a small fraction of Nrf2 escapes and enters the nucleus, polymerizes with small musculoaponeurotic fibrosarcoma protein, recognizes and binds to ARE, and activates the expression of antioxidant enzymes and phase II detoxification enzymes, including NADPH oxidase 1, HO-1, and SOD, among others. These free-radical scavenger enzymes can participate in the antioxidant defense mechanism of the body. Hence, when the body is stimulated by excessive ROS, the separation of Nrf2 and Keap1 increases, and more Nrf2 dissociates into the nucleus. Antioxidant enzyme expression is upregulated, and antioxidant effects are increased (Zhang et al., 2010; Tu et al., 2019).

The gut is the first place where endogenous ROS react with food. There are a variety of microorganisms in the gastrointestinal tract of healthy people (Ballard and Towarnicki, 2020). These microbes comprise a complex and diverse ecosystem in the gut, known as the gut microbiota, and there are about 400 bacterial species in the colon (Koeth et al., 2013). Usually, the imbalance of homeostasis between the host and gut microbiota will lead to impaired bodily function and induction of various diseases, such as cardiac, metabolic, cancer, neurodegenerative, and gastrointestinal diseases. Gut microbiota have become a new area of interest in disease research. Increasingly, studies have shown that the diversity of intestinal microbiota is changed during aging. Additionally, the abundance of microbiota related to glycolysis and short-chain fatty acids (SCFAs) is decreased and of microbiota related to protein decomposition is increased (Nicholson et al., 2012). Recently, numerous studies have revealed that the gut microbiota are generally related to a healthy-host status. Verdam et al. (2014) found that reduced bacterial diversity and a decreased ratio of *Bacteroidetes/Firmicutes* were found in an obesity group. With increasing age, the relative abundances of *Faecalibacterium*, *Bacteroidaceae*, and *Lachnospiraceae* were reduced (Badal et al., 2021).

It remains unclear if enhancement of the anti-aging effect of PS is directly related to the Keap1/Nrf2/ARE signaling pathway. Therefore, elucidation of the mechanism underlying the anti-aging effect of PS before and after processing would have great theoretical significance and potential clinical applications. We assumed that the chemical constitution of PS was changed by processing to produce greater antioxidant activity and that WPS has a positive effect on aging by regulating the gut microbiota and eliminating ROS stress by activating the Keap1/Nrf2/ARE pathway. The study aim was to investigate the anti-aging mechanism of PS and its wine steaming processed product in mice that focuses on the effect of D-gal surrounding the intestinal flora and on the Keap1/Nrf2/ARE pathway.

## 2 Materials and methods

### 2.1 Preparation of the RPS and WPS water exactions

RPS (Lot No.20210301) purchased from Shun Quanlong pharmaceutical Co., Ltd. met the Chinese Pharmacopoeia regulations (2020 edition, Part One) and was identified by Prof. Jun Wan (Southwest Jiaotong University, China) who conducts TCM research. Yellow rice wine (Lot No. 201812138) was purchased from Shandong Jimo yellow winery Co., Ltd. WPS was prepared in accordance with the Chinese Pharmacopeia’s processing standards (2020 edition, Part Four) and the steaming method. Following previous experiments, WPS was obtained by adding 20-g of wine to 100-g of RPS, soaking for 1h, steaming for 7h, braising for 3h, and drying at 60°C–80°C until the material did not stick to the hand (Shen et al., 2023).

According to the dose of clinical application per body surface area and the previous study (Liu, 2018), different concentrations of RPS and WPS was set (5 g/kg/d, 10 g/kg/d, 15 g/kg/d). Based on the volume of intragastric administration for mice as 0.1 mL/10 g, the corresponding solution concentrations were 0.5 g/mL, 1.0 g/mL, and 1.5 g/mL. One hundred grams of RPS or WPS botanical drugs were soaked in 600 mL of water at room temperature for 30 min, heating (decocting) at 100°C for 40 min, and filtrated by gauze. The residue was continuously boiled in 400 mL of water by repeating the decoction steps twice. Three filtrates were combined and concentrated to 200 mL with heating at 100°C, to get 0.5 g/mL the final concentration of crude drug. Then, the crude drug of 1.0 g/mL and 1.5 g/mL was separately made by continual heating up 0.5 g/mL concentration of crude drug to reduce the liquid by double and treble. The water extract was stored at 4°C for next use and prepared once a week.

### 2.2 Reagents

5-hydroxymethylfurfural (5-HMF) standard (MUST-21121510, Purity: 99.89%) and adenosine standard (MUST-21070613, Purity: 99.79%) were purchased from Chengdu Mansite Biotechnology Co., Ltd. (Sichuan, China). D-gal (P751206, Purity: 98.00%) was obtained from Chengdu Huaxia Chemical Reagent Co., Ltd. (Sichuan, China). Vitamin E (VitE, 2020121203, Purity: ≥50.00%) and anhydrous ethanol were provided by Chengdu Kelong Chemical Reagent Co., Ltd. (Sichuan, China). A hematoxylin-eosin (HE) staining kit (DH0020) was obtained from Leagene Biotechnology Co., Ltd. (Beijing, China). Biochemical kits for SOD, MDA, and GSH-PX were supplied by the Nanjing Jiancheng Institute of Biotechnology (Nanjing, China).

A BCA Protein Concentration Assay Kit (BL521A), RIPA cracking liquid (BL504A), phenylmethanesulfonyl fluoride (100 mM, BL507A), and 5×protein buffer (BL502B) were provided by Labgic Technology Co., Ltd. (Anhui, China). An Animal Total RNA Isolation Kit (RE-03014) was obtained from Chengdu Foregene Biotechnology Co., Ltd. (Sichuan, China). Beta actin (AF7018) and GAPDH (AF7018) were purchased from Affinity Biosciences (Jiangsu, China). HO-1 (E-AB-18231) and Keap1 (E-AB-19309) polyclonal antibodies were obtained from Elabscience Biotechnology Co., Ltd. (Hubei, China). Nrf2 polyclonal antibody (YT3189) was obtained from Immunoway Biotechnology Company (Texas, America). Goat anti-rabbit IgG HRP (70-GAR0072) and Goat anti-mouse IgG HRP (70-GAM0072) were provided by MultiSciences Biotech Co., Ltd. (Zhejiang, China).

HPLC/MS-grade acetonitrile was purchased from Sigma (Indiana, USA), and analytical-grade methanol and formic acid were supplied by Chengdu Jinshan Chemical Reagent Co., Ltd. (Sichuan, China).

A Research BIOMICS™ DNA Microprep Kit (Zymo, D4301), Clean Gel Recovery Kit (Zymo, D4008), KOD-Plus-Neo DNA PoKlymerase (TOYOBO, KOD-401B), and Hiseq Rapid SBS Kit v2 (Illmina, FC-402-4023 500 Cycle) were used in 16S rDNA sequencing of intestinal microbiota.

### 2.3 UPLC-Q-orbitrap HRMS analysis materials

UPLC-Q-Orbitrap HRMS was used to detect and identify the chemical composition of RPS and WPS in detail. The 1 g/mL water extracts of RPS and WPS were diluted to 20 mg/mL with methanol. A 50 μg/mL mixed standard solution of 5-HMF and adenosine was prepared in methanol. The sample and standard solutions were filtered through 0.22-μm filters and transferred into 1.5-mL sample bottles before analysis.

UPLC analysis was performed using a Thermo Fisher Scientific Vanquish with an ACQUITY UPLC BEH C_18_ column (2.1 mm × 50 mm, 1.7 μm) At a column temperature of 35°C. Mobile phase A was 0.1% formic acid and mobile phase B was acetonitrile. Gradient elution was performed as follows: 0–0.01 min, 5%B; 0.01–6.5 min, 5%–20%B; 6.5–13 min, 20%–28%B; 13–23 min, 28%–50%B; 23–33 min, 50%–65%B; 33–38 min, 65%–95%B; 38–40 min, 95%B. The flow rate was 0.3 mL/min, and the injection volume was 3 μL.

Mass spectrometry was performed using positive- and negative-ion modes from m/z 100–1500. An electrospray ion source and a spray voltage of 3.5 kV were used. Sheath gas and auxiliary gas flow rates were set at 35 arb and 10 arb, respectively. The ion source and auxiliary gas heating temperature was 350 °C. A full MS/data-dependent MS^2^ scan was used at two resolution levels of 35,000 and 17,500. The collision energy was set to three gradient levels of 20, 40, and 60 eV.

### 2.4 Experimental design for the animals

#### 2.4.1 Animals

Specific pathogen-free-grade male Kunming mice (six to seven weeks old weighing 18–22 g) were purchased from Chengdu Dashuo Experimental Animal Co., Ltd. (Sichuan, China, certificate number of SCXK [Chuan] 2020-030). All procedures were performed in accordance with the Implementation Rules for the Management of Medical Laboratory Animals (1988 edition) promulgated by the Ministry of Health and were approved by the Animal Experimentation Ethics Committee of Southwest Jiaotong University (No. SWJTU-2010-001). The mice were permitted free access to drinking water and diet and housed in cages at a temperature of 20°C–25°C, relative humidity of 30%–70%, and a 12 h light/dark cycle.

#### 2.4.2 Establishment of an aging model and drug intervention

After feeding in a week to adapt the environment, the mice were randomly divided into nine groups (10 mice per group) as follows: (i) control group (healthy controls, CG), (ii) model group (only D-gal, MG), (iii) positive group (D-gal + VitE [200 mg/kg/day], PG), (iv) RPS low-dose group (D-gal + RPS [5 g/kg/day], RPSG-L), (v) RPS medium-dose group (D-gal + RPS [10 g/kg/day], RPSG-M), (vi) RPS high-dose group (D-gal + RPS [15 g/kg/day], RPSG-H), (vii) WPS low-dose group (D-gal + WPS [5 g/kg/day], WPSG-L), (viii) WPS medium-dose group (D-gal + WPS [10 g/kg/day], WPSG-M), and (ix) WPS high-dose group (D-gal + WPS [15 g/kg/day], WPSG-H).

The aging model was established according to a method described previously (Zhao et al., 2021). The mice were intraperitoneally injected (i.p.) with D-gal at a dose of 500 mg/kg/day (0.1 mL/10 g) for 7 weeks, except for the mice in the CG who received an equal volume of 0.9% normal saline. From 14 days, the mice in the RPSG or WPSG were orally treated with different concentrations of the water extracts, and a gavage volume of 0.1 mL/10 g was used. For PG, based on the clinical administration dose per body surface area and combined with a previous study experience, the mice were administered VitE intragastrically (200 mg/kg/day, 0.1 mL/10 g). The same dose (0.1 mL/10 g) of normal saline was administered to the CG and MG. All groups were administered their designated solutions once a day for 32 days. The mental state and body weight of all mice were recorded regularly and dosage was adjusted by body weight.

### 2.5 Pathological examination

Pathological examination of the mice brain tissue was performed using HE staining. The brain tissue was divided into two parts. The left tissue was placed in 10% formaldehyde solution for 48 h to fix and then dehydrated, embedded in paraffin, and sliced. The slices (3 μm) were stained with an HE kit, sealed with neutral gum, and observed under histopathological conditions by a digital slice scanner. The right tissue was equally sliced in triplicate with a blade that was transferred and stored in liquid nitrogen for the following tests.

### 2.6 Index of immune organs

After the last administration, the mice were deprived of food and water for 12 h and their weights recorded. Subsequently, the mice were euthanized by dislocation of the spine, and tissue samples from the liver and spleen were quickly removed and rinsed with chilled saline. The surface water on the tissue was dried with filter paper. The organ weight was recorded, and the organ index was calculated as follows:

$$\text{Organ index}=\text{organ weight }\left(\text{mg}\right)\;/\text{body weight }\left(g\right)$$

### 2.7 Biochemical tests

Fresh blood was collected from the eyeballs of the mice. After placed for half an hour at room temperature, the serum was separated by centrifugation at 4°C and 3500 rpm for 10 min. The supernatant was collected separately and stored at −80°C. The slices of right-brain tissue used for biochemical tests were randomly selected. The activities of SOD, MDA, and GSH-PX in serum and brain tissue were determined using the appropriate biochemical kit in accordance with the manufacturer’s instructions. Absorbance of the sample was measured by a UV-VIS spectrophotometer (756, Yoke Instrument Co., Ltd.) (Shanghai, China) at a specific wavelength: 550 nm (SOD), 532 nm (MDA), and 412 nm (GSH-PX).

### 2.8 RT-PCR analysis

A randomly selected part of the right-brain tissue was used for RT-PCR analysis. Total RNA was extracted from the brain homogenate using an Animal Total RNA Isolation Kit. Reverse transcription into cDNA was performed using the 5× All-In-One MasterMix (with AccuRT Genomic DNA Removal kit). cDNA was amplified by EvaGreen Express 2×qPCR MasterMix-No Dye and quantitatively analyzed on an automatic medical PCR analysis system (Shanghai Hongshi Medical Technology Co., Ltd., China). The expressions of Keap1, HO-1, and Nrf2 were normalized to that of NADPH. Primer sequences for RT-PCR are shown in Table 1.

**TABLE 1:** Primer sequences for RT-PCR.

Gene	Primer sequences (5′–3′)	Product size (bp)
GAPDH	F: GGT​TGT​CTC​CTG​CGA​CTT​CA	183
	R: TGG​TCC​AGG​GTT​TCT​TAC​TCC	
Keap1	F: CGG​GGA​CGC​AGT​GAT​GTA​TG	85
	R: TGT​GTA​GCT​GAA​GGT​TCG​GTT​A	
HO-1	F: GAT​AGA​GCG​CAA​CAA​GCA​GAA	111
	R: CAG​TGA​GGC​CCA​TAC​CAG​AAG	
Nrf2	F: TAG​ATG​ACC​ATG​AGT​CGC​TTG​C	153
	R: GCC​AAA​CTT​GCT​CCA​TGT​CC	

The PCR amplification was performed as follows: a pre-denaturation step at 95°C for 10 min, a denaturation step at 95°C for 10 s, annealing extension at 60°C for 30 s. A total of 40 cycles were run. The mRNA levels of Keap1, Nrf2, and HO-1 mRNA were calculated by analyzing the threshold cycle (Ct) values of the samples. The ∆∆CT method was used to calculate the expressions normalized to NADPH. The 2^−ΔΔCT^ method was used to calculate the relative ratio of the target gene.

### 2.9 WB analysis

A part of the right-brain tissue used for WB analysis was randomly selected. Total protein from the brain tissue was extracted in RIPA lysis buffer. The lysate was transferred into a 1.5-mL centrifuge tube and centrifuged at 12,000 rpm, 4°C for 10 min. The supernatant was collected. Subsequently, the bicinchoninic acid (BCA) method was used to quantify the protein concentrations. Protein samples were separated by sodium dodecyl sulfate polyacrylamide gel electrophoresis gel and transferred into polyvinylidene fluoride membranes. The membranes were sealed with 5% defatted milk for 1 h at room temperature, incubated at 4°C overnight with primary antibodies against Keap1 (1:1000), Nrf2 (1:2000), HO-1 ((1:1000), and β-actin (1:5000) and washed three times (every 5 min) with tris buffer solution tween (TBST). After incubation with secondary antibodies at room temperature for 1 h, the membranes were washed with TBST three times (every 5 min). The bands were imaged, captured, and analyzed with Shanghai Qinxiang Chemical Analysis Software. The levels of protein expression normalized to β-actin were calculated.

### 2.10 16S rRNA gene sequencing of intestinal flora

Fresh mice feces were collected in sterile frozen tubes under sterile conditions and stored at −80°C. The total DNA of each sample was extracted and purified on the basis of the instructions of the Zymo Research BIOMICS DNA Miniprep Kit. Subsequently, DNA integrity was detected by 0.8% agarose gel electrophoresis, and the nucleic acid concentration was determined using the PicoGreen dye method. According to the sequencing region, the specific primers were synthesized to amplify the bacterial V4 variable regions of 16S rRNA genes, and the sequences for amplification were as follows: 515F (5′-GTGYCAGCMGCCGCGGTAA-3′) and 806R (5′-GGACTACHVGGGTWTCTAAT-3′). The PCR amplified process was as follows: a pre-denaturation step at 94°C for 1 min (1 cycle), a denaturation step at 94°C for 20 s, annealing at 54°C for 30 s and extension at 72°C for 30 s (25–30 cycle) and 72°C for 5 min (1 cycle). Each sample was repeatedly subjected to the above process three times, and PCR products in the linear phase were equally mixed for subsequent library construction. The target fragments of the PCR products were detected by 2% agarose gel electrophoresis, recycled using a Zymo clean Gel Recovery Kit, and qualified with Qubit@ 2.0 Fluorometer (Thermo Scientific). The library was constructed using the NEBNext Ultra II DNA Library Prep Kit for Illumina. Finally, high-throughput sequencing for amplified products was performed using an Illumina Hiseq Rapid SBS Kit v2 by Rhonin Biosciences (Chengdu, China).

### 2.11 Statistical analysis

Statistical analysis was performed using IBM SPSS Statistics 26.0 (IBM SPSS Statistics for Windows, IBM Corp., Armonk, NY) and GraphPad Prism 8.0 software (GraphPad Software, San Diego, CA). All data are shown as the mean ± standard deviation. Comparisons between multiple groups were assessed using one-way analysis of variance. Data of the group satisfying homogeneity of variance were then subjected to the least significant difference test, otherwise, the Tamhane T2 test was performed. Data were considered statistically significant at *p* < 0.05.

## 3 Results

### 3.1 Chemical composition

**FIGURE 1:**
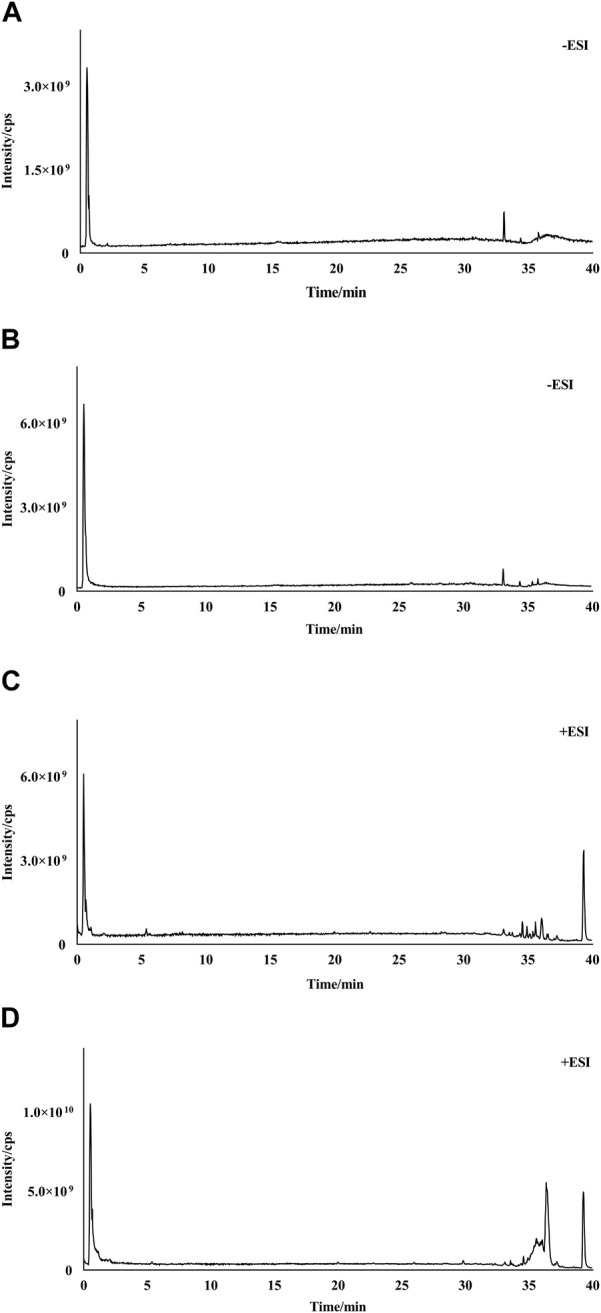
Total-ion chromatograms (TICs) of RPS and WPS water extraction. **(A)** TIC of RPS in negative-ion mode; **(B)** TIC of WPS in negative-ion mode; **(C)** TIC of RPS in positive-ion mode; **(D)** TIC of WPS in positive-ion mode.

The UPLC-Q-Orbitrap HRMS chromatograms of RPS and WPS water extraction in the negative- and positive-electrospray ion modes are shown in Figure 1. The Compound Discovery 3.0 and Xcalibur 2.0 programs were used to screen and process the spectrometry data. Using a mass error standard of <5 ppm between the theoretical and experimental data, the compositions of RPS and WPS were determined by high-precision measurement of the precursor and product ions. A total of 52 compounds of RPS and WPS were identified according to standards, mass spectrometry fragment modes, mass spectrometry libraries (including mzCloud datebase, mzVault datebase and Massbank datebase) and relative references. As shown in Table 2, there were a significant differences in compound type and content between RPS and WPS, with 42 compounds in RPS and 46 compounds in WPS. Succinic acid, 5′-S-methyl-5′-thioadenosine, indole-3-acrylic acid, azelaic acid, sakuranetin, and luteolin were detected only in RPS, whereas DL-malic acid, 5-hydroxymethyl-2-furaldehyde, deoxyribose, 2-furoic acid, δ-gluconic acid δ-lactone, nobiletin, 3-n-butylphathlide, ursolic acid, 16-hydroxyhexadecanoic acid, and 1-stearoylglycerol were detected only in WPS.

**TABLE 2:** UPLC-Q-Orbitrap HRMS data of the characterized compounds in RPS and WPS.

No	Chemical name	Formula	Error(δ)/ppm	Detected Mass (m/z) ion type	RT/min	MS fragments	R	W	Fold change
1	Sucrose	C_12_H_22_O_11_	−2.64	341.10898 [M−H]^−^	0.476	89.02345, 71.01282, 59.01281	+	+	−0.27
2	DL-Arginine	C_6_H_14_N_4_O_2_	−0.01	175.11879 [M + H]^+^	0.49	116.07079, 70.06573, 60.05629	+	+	−0.46
3	Choline	C_5_H_13_NO	0.02	104.10725 [M + H]^+^	0.492	87.04451, 60.08141	+	+	0.01
4	4-AMINOBUTANOATE	C_4_H_9_NO_2_	3.52	104.10721 [M + H]^+^	0.497	60.08141	+	+	−0.55
5	D-(−)-Fructose	C_6_H_12_O_6_	−0.03	179.05556 [M−H]^−^	0.498	89.02342, 71.01282, 59.01279	+	+	3.87
6	Adenosine	C_10_H_13_N_5_O_4_	0	268.1041 [M + H]^+^	0.506	136.0618	+	+	0.65
7	Adenine	C_5_H_5_N_5_	0	136.06171 [M + H]^+^	0.512	119.03548	+	+	0.28
8	L-Homoserine	C_4_H_9_NO_3_	0.01	120.06563 [M + H]^+^	0.516	74.06061, 56.05016	+	+	1.39
9	D-(+)-Proline	C_5_H_9_NO_2_	0.01	116.07075 [M + H]^+^	0.52	70.06571	+	+	−0.28
10	DL-Malic acid	C_4_H_6_O_5_	−0.06	133.01343 [M−H]^−^	0.521	115.00276, 71.01279	−	+	−
11	2-Hydroxyphenylalanine	C_9_H_11_NO_3_	0	182.08119 [M + H]^+^	0.53	136.07561, 123.04416	+	+	0.74
12	5-Hydroxymethyl-2-furaldehyde	C_6_H_6_O_3_	0.01	127.0391 [M + H]^+^	0.584	109.02828, 81.03403, 69.03411	−	+	−
13	Citric acid	C_6_H_8_O_7_	−0.02	191.01926 [M−H]^−^	0.679	111.00788, 87.00788, 85.02851	+	+	2.65
14	4-Oxoproline	C_5_H_7_NO_3_	−0.06	128.03452 [M−H]^−^	0.683	82.02883	+	+	−0.07
15	L-Pyroglutamic acid	C_5_H_7_NO_3_	0.02	130.05009 [M + H]^+^	0.685	84.04488	+	+	0.08
16	DEOXYRIBOSE	C_5_H_10_O_4_	−0.06	133.04982 [M−H]^−^	0.686	115.00275, 71.01279	−	+	−
17	2-Furoic acid	C_5_H_4_O_3_	−0.08	111.00792 [M−H]^−^	0.691	67.01788	−	+	−
18	Succinic acid	C_4_H_6_O_4_	−0.06	117.01862 [M−H]^−^	0.749	116.92767, 99.92487, 73.02848	+	−	−
19	DL-Norleucine	C_6_H_13_NO_2_	0.01	132.10201 [M + H]^+^	0.766	86.09688	+	+	−0.16
20	δ-Gluconic acid δ-lactone	C_6_H_10_O_6_	−0.02	177.04015 [M−H]^−^	0.947	129.01849, 99.00777, 71.01278	−	+	−
21	L-Phenylalanine	C_9_H_11_NO_2_	0	166.08632 [M + H]^+^	1.072	120.08093	+	+	0.38
22	Maltol	C_6_H_6_O_3_	0.02	127.03922 [M + H]^+^	1.149	109.02870, 81.03404	+	+	37.31
23	5′-S-Methyl-5′-thioadenosine	C_11_H_15_N_5_O_3_S	0	298.09686 [M + H]^+^	1.842	136.06171	+	−	−
24	Indole-3-acrylic acid	C_11_H_9_NO_2_	0	188.07062 [M + H]^+^	2.054	146.05997, 118.06531	+	−	−
25	Acetanilide	C_8_H_9_NO	0.01	136.07585 [M + H]^+^	3.404	69.0341	+	+	0.77
26	Dibenzylamine	C_14_H_15_N	0.01	198.12787 [M + H]^+^	5.23	91.0547	+	+	0.33
27	N-Feruloyloctopamine	C_18_H_19_NO_5_	0	330.13354 [M + H]^+^	6.028	310.10880, 161.02376	+	+	0.28
28	Azelaic acid	C_9_H_16_O_4_	−0.02	187.0972 [[M−H]^−^	6.83	125.09634	+	−	−
29	Sakuranetin	C_16_H_14_O_5_	0	287.09137 [M + H]^+^	6.906	167.03377	+	−	−
30	Luteolin	C_15_H_10_O_6_	0.01	285.04071 [M−H]^−^	9.117	133.02869	+	−	−
31	9-Oxo-ODE	C_18_H_30_O_3_	0	295.22687 [M + H]^+^	15.598	277.21628, 81.07051, 67.05489	+	+	−0.13
32	Nobiletin	C_21_H_22_O_8_	0	403.13889 [M + H]^+^	18.022	373.09167	−	+	−
33	3-n-Butylphathlide	C_12_H_14_O_2_	0	191.10666 [M + H]^+^	19.333	173.09607, 145.10110	−	+	−
34	Tangeritin	C_20_H_20_O_7_	0	373.12830 [M + H]^+^	20.041	343.08096	+	+	0.28
35	Bis(4-ethylbenzylidene)sorbitol	C_24_H_30_O_6_	0	415.21161 [M + H]^+^	22.746	119.08575	+	+	1.65
36	(+/−)12 (13)-DiHOME	C_18_H_34_O_4_	0.01	313.23883 [M−H]^−^	22.801	183.13852, 129.09145, 99.08057	+	+	0.54
37	Nootkatone	C_15_H_22_O	0	219.17438 [M + H]^+^	23.674	111.08067, 109.10144	+	+	4.96
38	Dodecyl sulfate	C_12_H_26_O_4_S	0.01	265.14804 [M−H]^−^	25.965	96.95911	+	+	1.28
39	Linoleoyl ethanolamide	C_20_H_37_NO_2_	0	324.28964 [M + H]^+^	30.213	62.06072	+	+	−0.06
40	4-Dodecylbenzenesulfonic acid	C_18_H_30_O_3_S	0.01	325.18448 [M−H]^−^	30.251	183.01152	+	+	2.41
41	Myristyl sulfate	C_14_H_30_O_4_S	0.01	293.1796 [M−H]^−^	30.546	96.95915	+	+	1.15
42	Oleanolic acid	C_30_H_48_O_3_	0	457.3679 [M + H]^+^	32.042	203.17940, 191.17934, 95.08601	+	+	0.65
43	Ursolic acid	C_30_H_48_O_3_	0	457.3678 [M + H]^+^	32.046	411.36203, 163.14288, 95.08600	−	+	−
44	16-Hydroxyhexadecanoic acid	C_16_H_32_O_3_	0.01	271.22824 [M−H]^−^	32.175	222.22221	−	+	−
45	Hexadecanamide	C_16_H_33_NO	0	256.2634 [M + H]^+^	32.486	102.09168, 88.07610, 57.07059	+	+	0.14
46	4-Methoxycinnamic acid	C_10_H_10_O_3_	0	179.07028 [M + H]^+^	32.736	161.05968, 133.06483	+	+	0.05
47	Oleamide	C_18_H_35_NO	0	282.27921 [M + H]^+^	32.98	72.08139	+	+	0.1
48	Stearamide	C_18_H_37_NO	0	284.29477 [M + H]^+^	33.765	116.10732	+	+	−0.95
49	Stearoyl Ethanolamide	C_20_H_41_NO_2_	−0.01	328.3208 [M + H]^+^	33.83	62.0607	+	+	−0.28
50	Tridemorph	C_19_H_39_NO	−0.01	298.31024 [M + H]^+^	34.906	102.09168	+	+	−0.30
51	1-Stearoylglycerol	C_21_H_42_O_4_	−0.01	359.31525 [M + H]^+^	35.019	95.08588, 71.08614, 57.07056	−	+	−
52	Docosanamide	C_22_H_45_NO	0	340.35739 [M + H]^+^	36.16	284.29462	+	+	−0.65

Notes: RT, retention time; R, RPS; W, WPS; +, present; −, absent.

### 3.2 Influence of PS on the mental state and body weight in aging mice

Before modeling, the mice in each group had shown a good mental state, bright and smooth hair, normal diet and defecation, high activity, and quick responses. After establishing the aging model, the MG mice showed a poor mental state, yellow hair, lost hair, decreasing activity, listlessness, and clustering. The overall state in the MG was worse than that in the CG and other drug groups, and their weight increase slowed. The mice body weights in each group are shown in Table 3.

**TABLE 3:** Body weights (g) of the mice in each group (*n* = 10).

Group	Starting (i.p.) inject D-gal (g)		Starting intragastric administration (g)				
	0 d	7 d	14 d	21 d	28 d	35 d	42 d
CG	28.07 ± 1.33	31.14 ± 1.38	33.05 ± 1.40	35.74 ± 0.74	36.90 ± 1.54	38.01 ± 1.05	38.78 ± 1.08
MG	28.63 ± 1.34	29.76 ± 1.56	31.42 ± 1.76	32.15 ± 1.32^**^	33.17 ± 2.57^**^	33.78 ± 2.54^**^	34.30 ± 2.05^**^
PG	29.05 ± 1.35	30.89 ± 1.45	32.46 ± 2.44	34.66 ± 2.02	36.08 ± 1.91^##^	37.38 ± 1.17^##^	38.53 ± 1.30^##^
RPSG-L	28.23 ± 1.23	29.93 ± 1.75	31.78 ± 1.85	33.31 ± 2.06	34.95 ± 1.86^*^	35.74 ± 1.96^*#^	36.11 ± 1.63^**#^
RPSG-M	27.89 ± 1.43	29.50 ± 2.21	31.85 ± 2.26	33.63 ± 1.94	34.30 ± 2.40^**^	34.98 ± 2.63^**^	36.17 ± 1.85^**#^
RPSG-H	28.37 ± 1.80	29.70 ± 2.05	31.55 ± 1.90	32.74 ± 2.04^*^	34.55 ± 2.13^*^	35.12 ± 2.18^**^	35.69 ± 2.06^**^
WPSG-L	27.77 ± 1.37	29.63 ± 2.72	32.36 ± 1.87	33.84 ± 1.89	35.29 ± 2.06^#^	36.37 ± 2.35^##^	37.62 ± 2.02^##^
WPSG-M	28.21 ± 2.80	29.66 ± 2.69	32.21 ± 2.09	34.17 ± 2.99	35.83 ± 2.63^##^	37.3 ± 2.47^##∆^	38.14 ± 2.04^##∆^
WPSG-H	28.58 ± 1.35	30.20 ± 1.58	32.65 ± 1.72	34.87 ± 2.54	35.42 ± 1.60^#^	36.16 ± 1.81^#^	37.23 ± 2.28^##^

Notes: CG, healthy controls; MG, only D-gal; PG, D-gal + VitE (200 mg/kg/day); RPSG-L, D-gal + RPS (5 g/kg/day); RPSG-M, D-gal + RPS (10 g/kg/day); RPSG-H, D-gal + RPS (15 g/kg/day); WPSG-L, D-gal + WPS (5 g/kg/day); WPSG-M, D-gal + WPS (10 g/kg/day); WPSG-H, D-gal + WPS (15 g/kg/day). ^*^ *p* < 0.05, ^**^ *p* < 0.01, compared with the CG. ^#^ *p* < 0.05, ^##^ *p* < 0.01, compared with the MG. ^∆^ *p* < 0.05, ^∆∆^ *p* < 0.01, compared with the same dose of RPSG. ^&^ *p* < 0.05, ^and&^ *p* < 0.01, compared with the different dose of RPSG, or WPSG.

There was no significant difference in the mean body weights among the groups at the start of injecting D-gal. At 21 days, the body weights were significantly lower in the MG and RPSG-H than in the CG (*p* < 0.01 or *p* < 0.05). After 28 days, compared with the MG, body weight of mice in all doses WPSG was significantly increased (*p* < 0.01). After 35 days, the body weight of mice in the WPSG-M increased compared with that in the RPSG-M (*p* < 0.05). At 42 days, except of RPSG-H, the weights of all doses groups increased more rapidly than in the MG (*p* < 0.01 or *p* < 0.05), whereas there was no significant difference among different doses of RPS and WPS (*p* < 0.05).

### 3.3 Influence of PS on pathological changes in the brain tissue of aging mice

**FIGURE 2:**
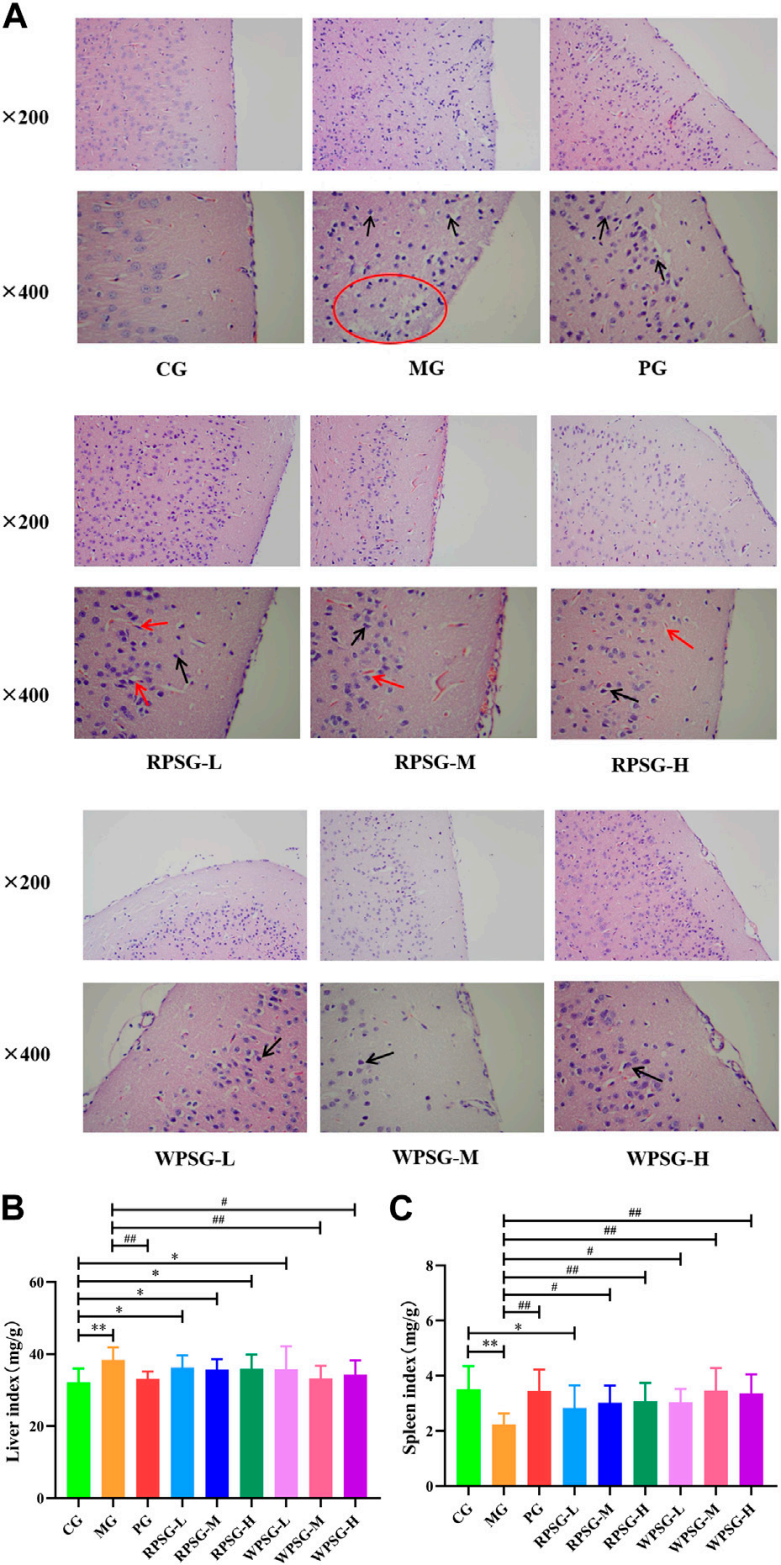
Therapeutic effects of RPS and WPS in the aging mice (n = 10). **(A)** Pathological examination of brain tissue (magnification ×200 and ×400); Neuronal cell size decreasing (black arrow); Formation of vacuoles/reticular structure (red arrow/circle); **(B)** Liver index; **(C)** Spleen index. CG, healthy controls; MG, only D-gal; PG, D-gal + VitE (200 mg/kg/day); RPSG-L, D-gal + RPS (5 g/kg/day); RPSG-M, D-gal + RPS (10 g/kg/day); RPSG-H, D-gal + RPS (15 g/kg/day); WPSG-L, D-gal + WPS (5 g/kg/day); WPSG-M, D-gal + WPS (10 g/kg/day); WPSG-H, D-gal + WPS (15 g/kg/day). ^*^ *p* < 0.05, ^**^ *p* < 0.01, compared with the CG. ^#^ *p* < 0.05, ^##^ *p* < 0.01, compared with the MG. ^∆^ *p* < 0.05, ^∆∆^ *p* < 0.01, compared with the same dose of RPSG. ^&^ *p* < 0.05, ^&&^ *p* < 0.01, compared with the different dose of RPSG or WPSG.

The state of neuronal cells was observed by H&E staining (Figure 2A). In the CG, the cortical brain tissue structure showed no pathological damage, which was evidenced by the clear contours of neurons and nucleoli, vacuolated nuclei, and intact nerve fibers and glial cells. In the MG, a large number of cortical neurons had shrunk, cell volumes had decreased, and the cerebral cortex was significantly thinned and appeared as a reticular structure. There were cavities around the nucleus and blood vessels, which showed severe edema. HE staining was enhanced and stained blue-purple. Compared with those in the MG, the lesions and edema degree of the PG, RPSG, and WPSG were reduced, and the cortical neurons showing slight swelling, cytoplasmic vacuoles, and unclear cell contours. These findings were consistent with the disease characteristics of aging caused by D-gal, indicating that the aging model was successfully replicated.

### 3.4 Influence of PS on the immune organ index in aging mice

The antioxidant capacity of the body is closely related to health status. As important immune and metabolic organs of the body, the spleen and liver indexes reflect the degree of cell proliferation and are closely related to the regulation of health functions. As is shown in Figure 2B, C, compared with those in the CG, the liver index of mice significantly increased and the spleen index significantly decreased in the MG (*p* < 0.01). After drug intervention, the liver index was significantly lower in the PG, WPSG-M, and WPSG-H than in the MG (*p* < 0.01 or *p* < 0.05) and was not significantly different from that in the CG (*p* > 0.05). The spleen indexes were significantly higher in all drug intervention groups than in the MG (*p* < 0.01 or *p* < 0.05) and restored to the level of the CG except for RPSG-L. The above results indicate that RPS and WPS could improve the immune organ index in aging mice, and WPS was more effective than RPS. There were no significant differences between different doses of RPS and WPS.

### 3.5 Influence of PS on biochemical activities in aging mice

**FIGURE 3:**
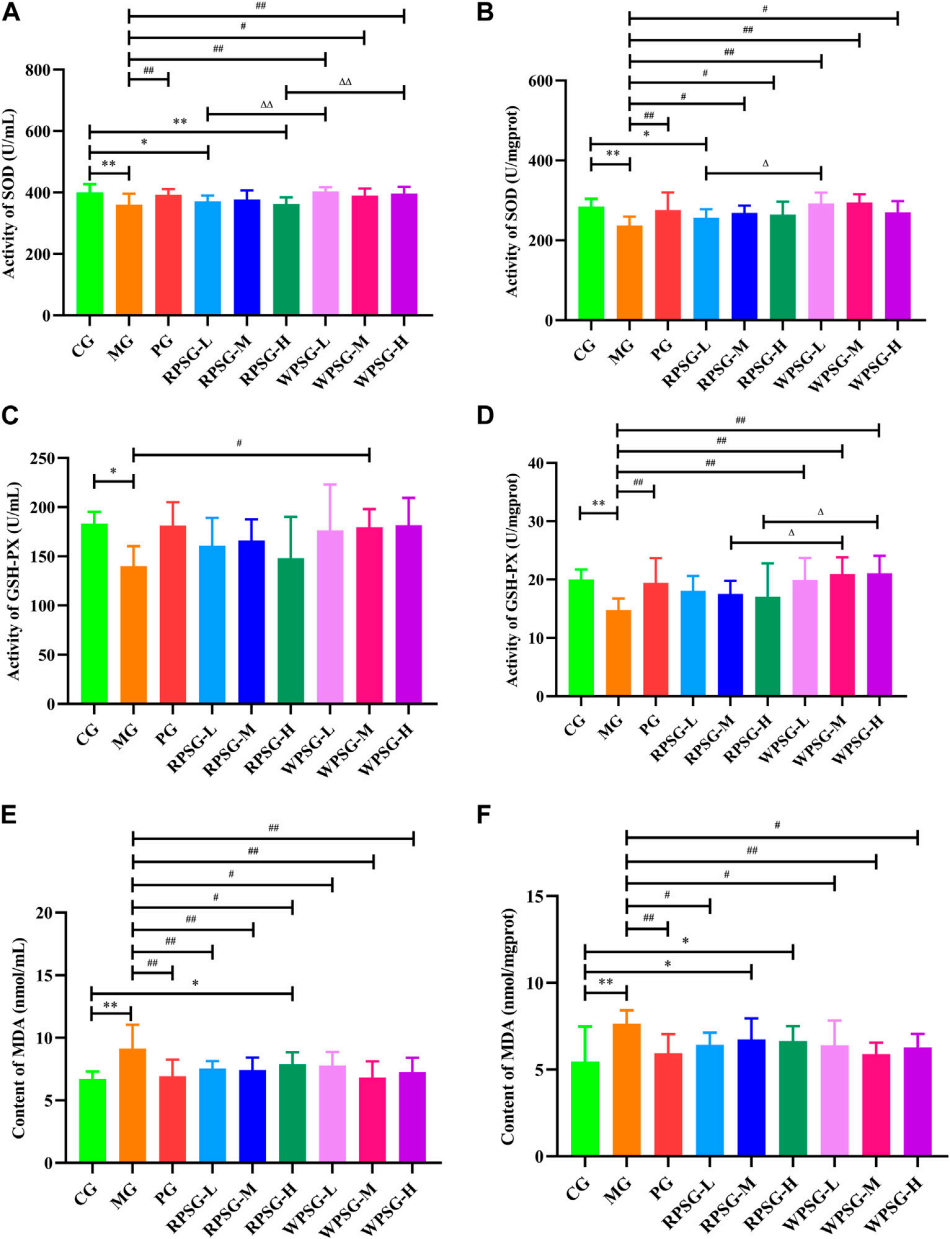
Biochemical activities of RPS and WPS in the aging mice (n = 8). **(A)** Activity of SOD in serum; **(B)** Activity of SOD in brain tissue; **(C)** Activity of GSH-PX in serum; **(D)** Activity of GSH-PX in brain tissue; **(E)** Activity of MDA in serum; and **(F)** Activity of MDA in brain tissue. CG, healthy controls; MG, only D-gal; PG, D-gal + VitE (200 mg/kg/day); RPSG-L, D-gal + RPS (5 g/kg/day); RPSG-M, D-gal + RPS (10 g/kg/day); RPSG-H, D-gal + RPS (15 g/kg/day); WPSG-L, D-gal + WPS (5 g/kg/day); WPSG-M, D-gal + WPS (10 g/kg/day); WPSG-H, D-gal + WPS (15 g/kg/day). ^*^ *p* < 0.05, ^**^ *p* < 0.01, compared with the CG. ^#^ *p* < 0.05, ^##^ *p* < 0.01, compared with the MG. ^∆^ *p* < 0.05, ^∆∆^ *p* < 0.01, compared with the same dose of RPSG. ^&^ *p* < 0.05, ^&&^ *p* < 0.01, compared with the different dose of RPSG or WPSG.

Compared with the CG, the expression levels of SOD (Figure 3A, B) and GSH-PX (Figure 3C, D) in the MG were remarkably decreased and the expression levels of MDA (Figure 3E, F) were notably increased (*p* < 0.01 or *p* < 0.05). The WPSG could adjust SOD, MDA, and GSH-PX to normal levels. RPSG could adjust SOD and MDA to normal levels. However, the regulatory effect was worse for RPSG than for WPSG. The results showed that WPS was superior to RPS for upregulating SOD and GSH-PX and lowering MDA. There were no significant distinctions between the different dose groups of RPS or WPS. According to the above and previous results, the medium-dose group of PS demonstrated the best therapeutic effect. Therefore, the RPSG-M and WPSG-M were considered as the default groups for the following tests to illustrate the pharmacodynamic mechanism.

### 3.6 Influence of PS on the Keap1//Nrf2/ARE signal pathway in the aging mice

**FIGURE 4:**
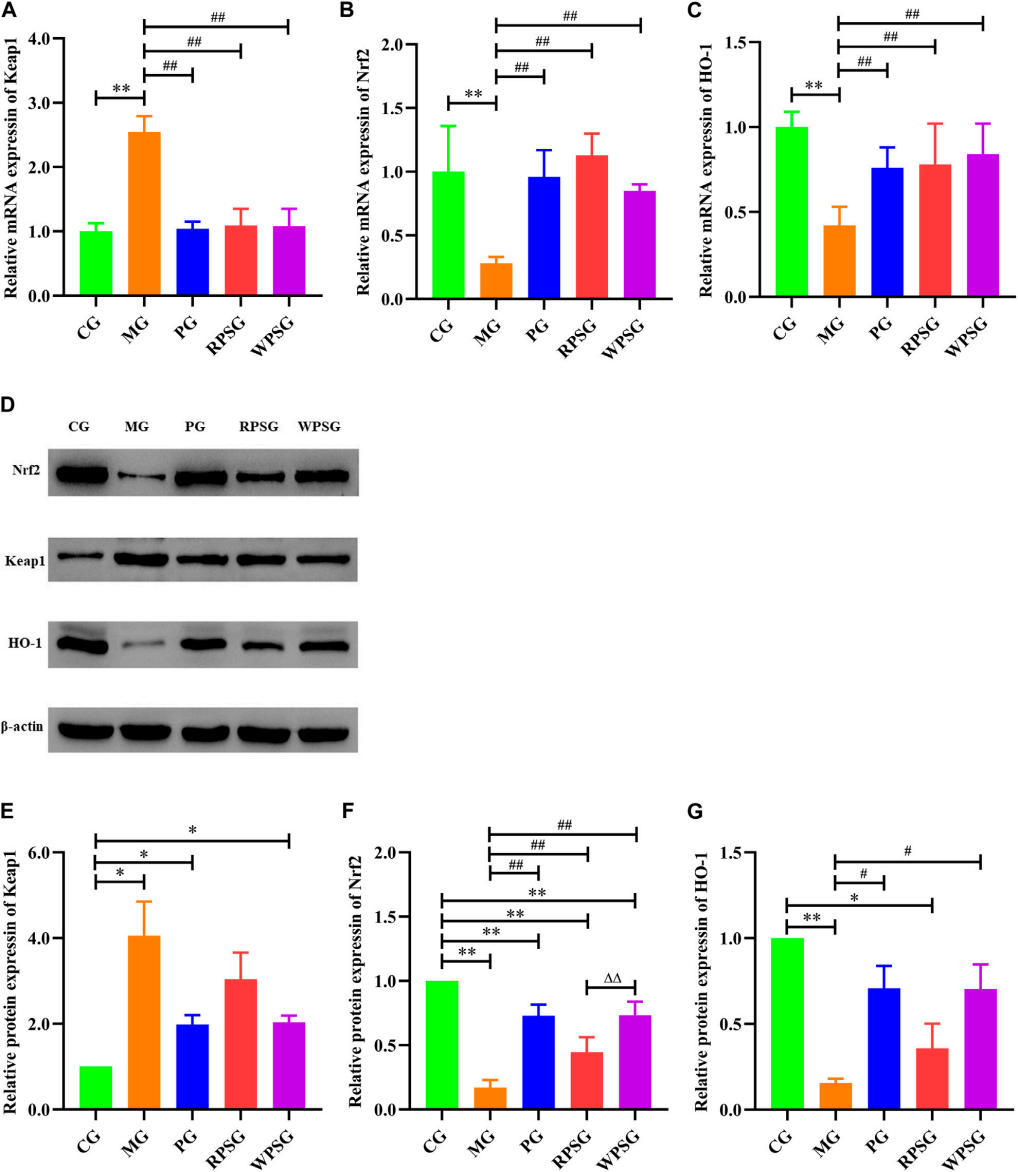
The effect of PS on the Keap1/Nrf2/ARE signal pathway in the brain tissue of aging mice (n = 4). **(A)** Relative mRNA expression of Keap1; **(B)** Relative mRNA expression of the Nrf2; **(C)** Relative mRNA expression of HO-1; **(D)** Associated protein bands in the Keap1/Nrf2/ARE signal pathway; **(E)** Relative protein expression of Keap1; **(F)** Relative protein expression of Nrf2; **(G)** Relative protein expression of HO-1. CG, healthy controls; MG, only D-gal; PG, D-gal + VitE (200 mg/kg/day); RPSG, D-gal + RPS (10 g/kg/day); WPSG, D-gal + WPS (10 g/kg/day). ^*^ *p* < 0.05, ^**^ *p* < 0.01, compared with the CG. ^#^ *p* < 0.05, ^##^ *p* < 0.01, compared with the MG. ^∆^ *p* < 0.05, ^∆∆^ *p* < 0.01, compared with the same dose of RPSG. ^&^ *p* < 0.05, ^&&^ *p* < 0.01, compared with the different dose of RPSG or WPSG.

RT-PCR was used to assess the mRNA expression of Keap1, Nrf2, and HO-1 in the brain tissue. As illustrated in Figure 4A–C, compared with the CG, the mRNA expressions of Nrf2 and HO-1 were remarkably decreased and that of Keap1 was remarkably increased in the MG (*p* < 0.01). The mRNA expression levels of Nrf2 and HO-1 were significantly upregulated in the RPSG, WPSG, and PG relative to those in the MG (*p* < 0.01).

WB was used to further check the Keap1, Nrf2, and HO-1 protein expressions in aging mice. As shown in Figure 4D–G, the protein expressions of Nrf2 and HO-1 were lower and that of Keap1 was higher in the MG than in CG (*p* < 0.01 or *p* < 0.05). Compared with the MG, the expression of Nrf2 was increased in the PG, RPSG, and WPSG, and the WPSG showed greater improvement than the RPSG (*p* < 0.01). The expressions of Keap1 and HO-1 in the PG and WPSG were significantly different from those in the MG (*p* < 0.05). Combined with the RT-PCR results, these results show that PS treatment significantly alleviated oxidative stress impairment in aging mice by improving the expressions of Nrf2 and HO-1 and suppressing the expression of Keap1 in the mRNA and protein levels. However, the level of CG was restored to a greater degree in the WPSG than in the RPSG.

### 3.7 Influence of PS on the intestinal flora in aging mice

#### 3.7.1 Alpha diversity analysis

**FIGURE 5:**
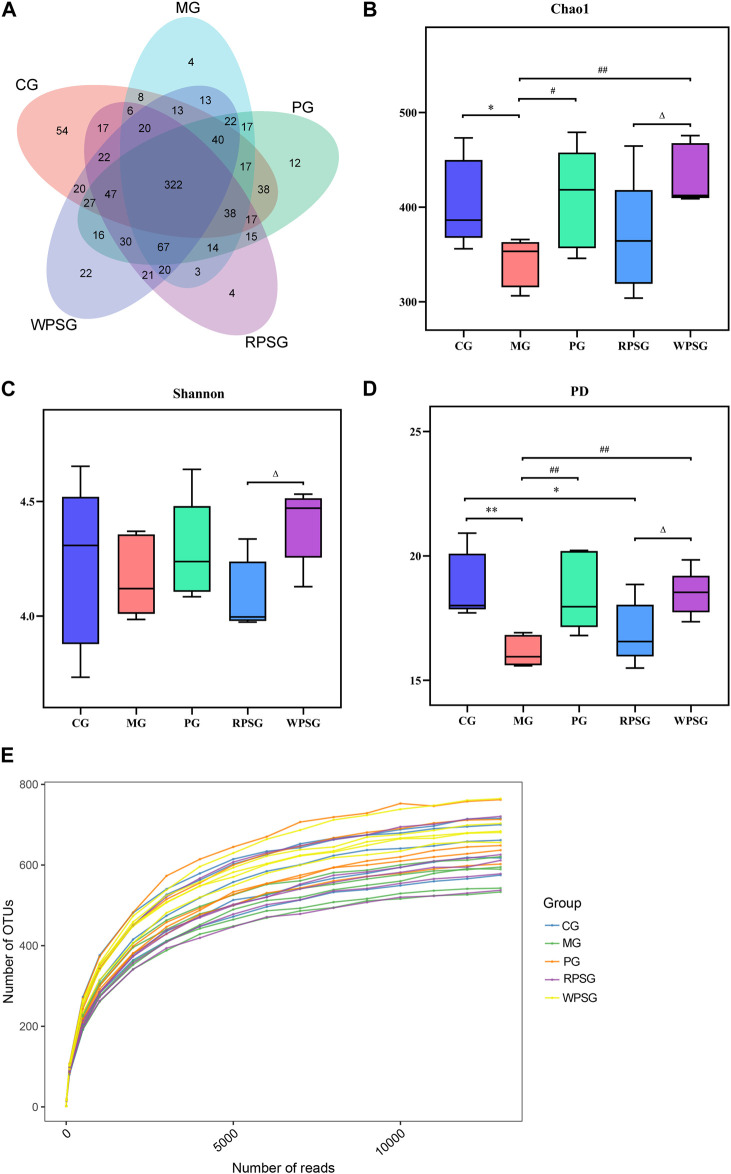
Alpha diversity of the intestinal microbiota (n = 5). **(A)** Venn diagram; **(B)** Chao1 index; **(C)** Shannon index; **(D)** PD index; and **(E)** Rarefaction curves. CG, healthy controls; MG, only D-gal; PG, D-gal + VitE (200 mg/kg/day); RPSG, D-gal + RPS (10 g/kg/day); WPSG, D-gal + WPS (10 g/kg/day). ^*^ *p* < 0.05, ^**^ *p* < 0.01, compared with the CG. ^#^ *p* < 0.05, ^##^ *p* < 0.01, compared with the MG. ^∆^ *p* < 0.05, ^∆∆^ *p* < 0.01, compared with the RPSG.

After removing the unqualified sequences, 30,152 effective tags were collected and 986 operational taxonomic units (OTUs) were obtained. A Venn diagram (Figure 5A) shows the overlap of OTUs among each group, and a similar relationship between different samples of each group was demonstrated. As shown in Figure 5E, the dilution curve tended to be flat with an increasing number of reads, whereas the number of OTUs hardly increased at all, indicating that sequencing could basically cover all species in the sample and that increasing the amount of data would only produce a small number of low-abundance species. The alpha diversity is used to characterize a species’ richness, evenness, and diversity of a sample. Typically, the Chao1 index is used to estimate the total number of species, the Shannon index is used to assess microbial diversity, and the PD index is used to measure phylogenetic diversity. The Chao1 and PD indexes were lower in the MG than in the CG (Figures 5B–D) and higher in the PG and WPSG than in the MG (*p* < 0.01 or *p* < 0.05). The Chao1, Shannon, and PD indexes were significantly different between the RPSG and WPSG (*p* < 0.05). These results indicated that the species’ richness and diversity were higher in the CG than in the MG, and the WPSG was more effective in improving the community diversity to normal levels than the RPSG.

#### 3.7.2 Beta diversity analysis

**FIGURE 6:**
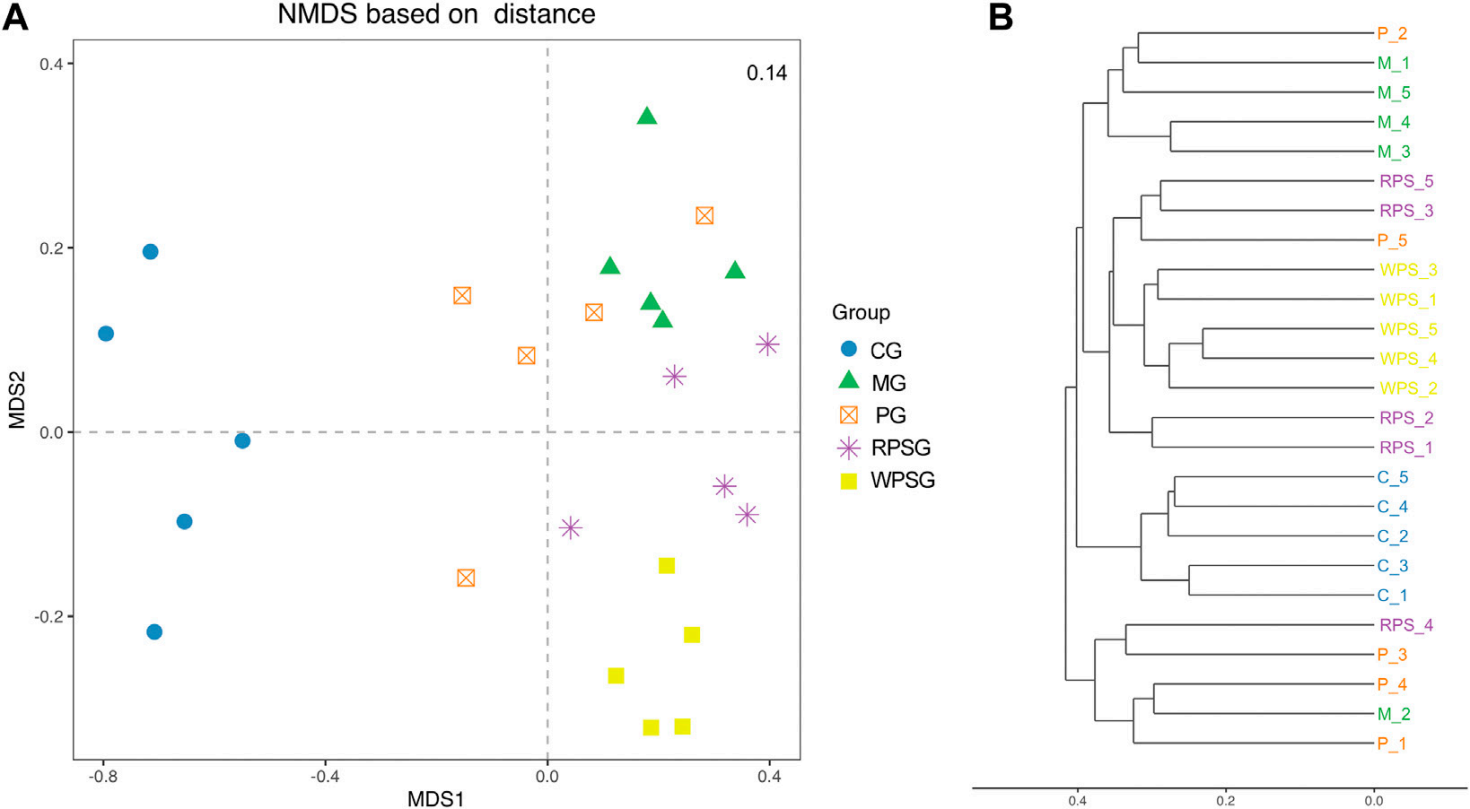
Beta diversity of the intestinal microbiota (n = 5). **(A)** NMDS analysis based on Bray–Curtis; **(B)** Clustering Dendrogram, unweighted. CG, healthy controls; MG, only D-gal; PG, D-gal + VitE (200 mg/kg/day); RPSG, D-gal + RPS (10 g/kg/day); WPSG, D-gal + WPS (10 g/kg/day).

Microbial community structures of different samples were assessed by Beta diversity analysis. UniFrac is one of the most common methods to analyze the distance between microbial communities, including the use of weighted and unweighted algorithms (Lozupone et al., 2011). Nonmetric multidimensional scaling (NMDS) results based on the Bray–Curtis distance matrix (Figure 6A), and the Unweighted Clustering Dendrogram (Figure 6B) were performed. The stress value was <0.2, indicating that the NMDS analysis results were reliable. The sample points between the CG and MG were completely separated, indicating that, compared with that of the CG, the intestinal microbiota composition of the MG changed significantly. Furthermore, at the MDS2 level, the PG, RPSG and WPSG showed different separation compared with the MG, besides, the WPSG showed greater separation from the MG than from the RPSG, which indicated that WPSG was more effective in adjusting the structure of the intestinal microbiota.

#### 3.7.3 Community composition analysis of the intestinal flora in aging mice

According to the abundance of OTU and the results of annotated taxonomic information, the dominant species with relative abundance at the phylum and genus levels were selected and examined by cumulative bar plot to explore the structural composition of the intestinal flora in aging mice.

**FIGURE 7:**
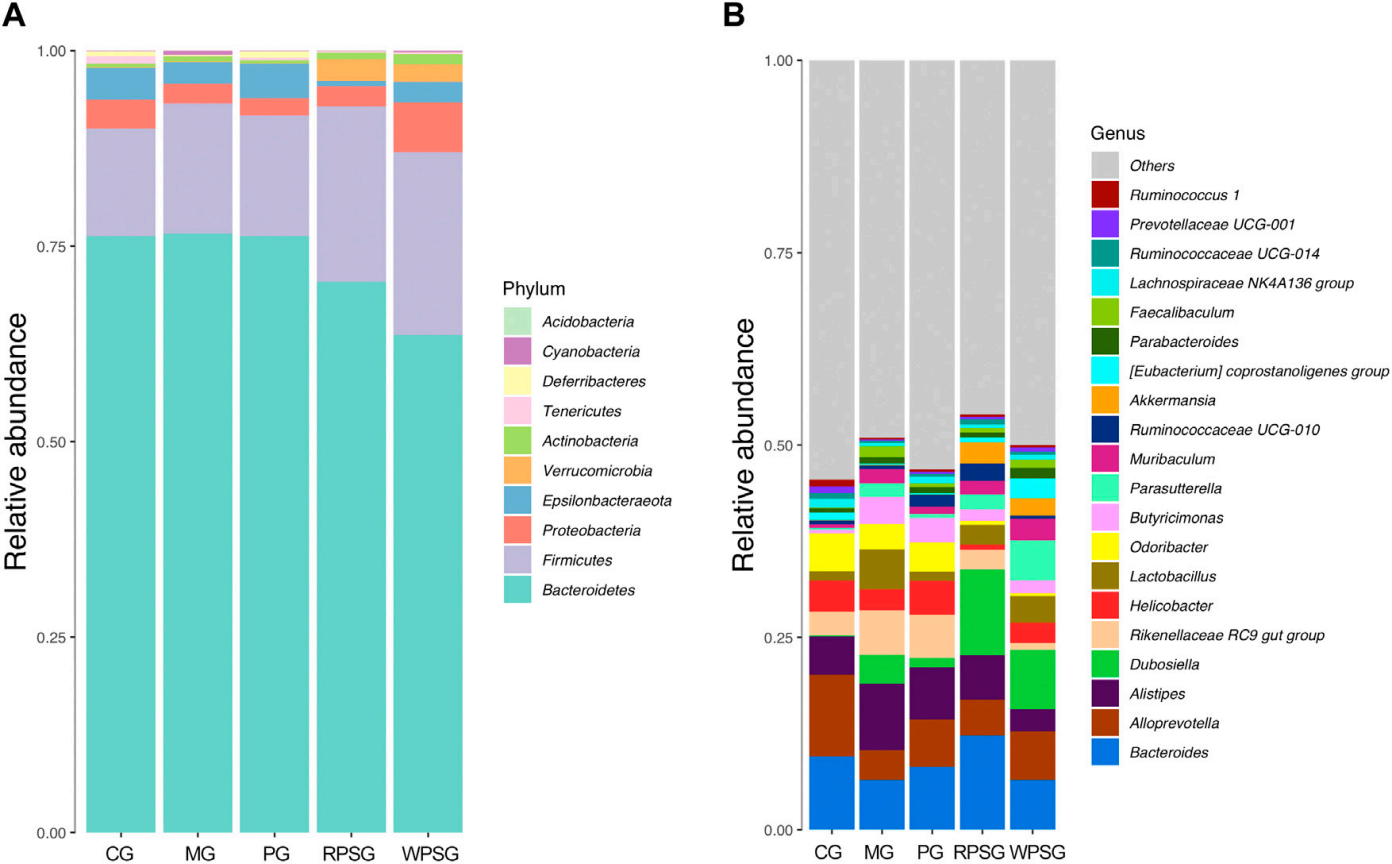
The community composition of intestinal flora in aging mice (n = 5). **(A)** Bar plot of the relative abundance at the phylum level; **(B)** Bar plot of the relative abundance at the genus level. CG, healthy controls; MG, only D-gal; PG, D-gal + VitE (200 mg/kg/day); RPSG, D-gal + RPS (10 g/kg/day); WPSG, D-gal + WPS (10 g/kg/day).

**FIGURE 8:**
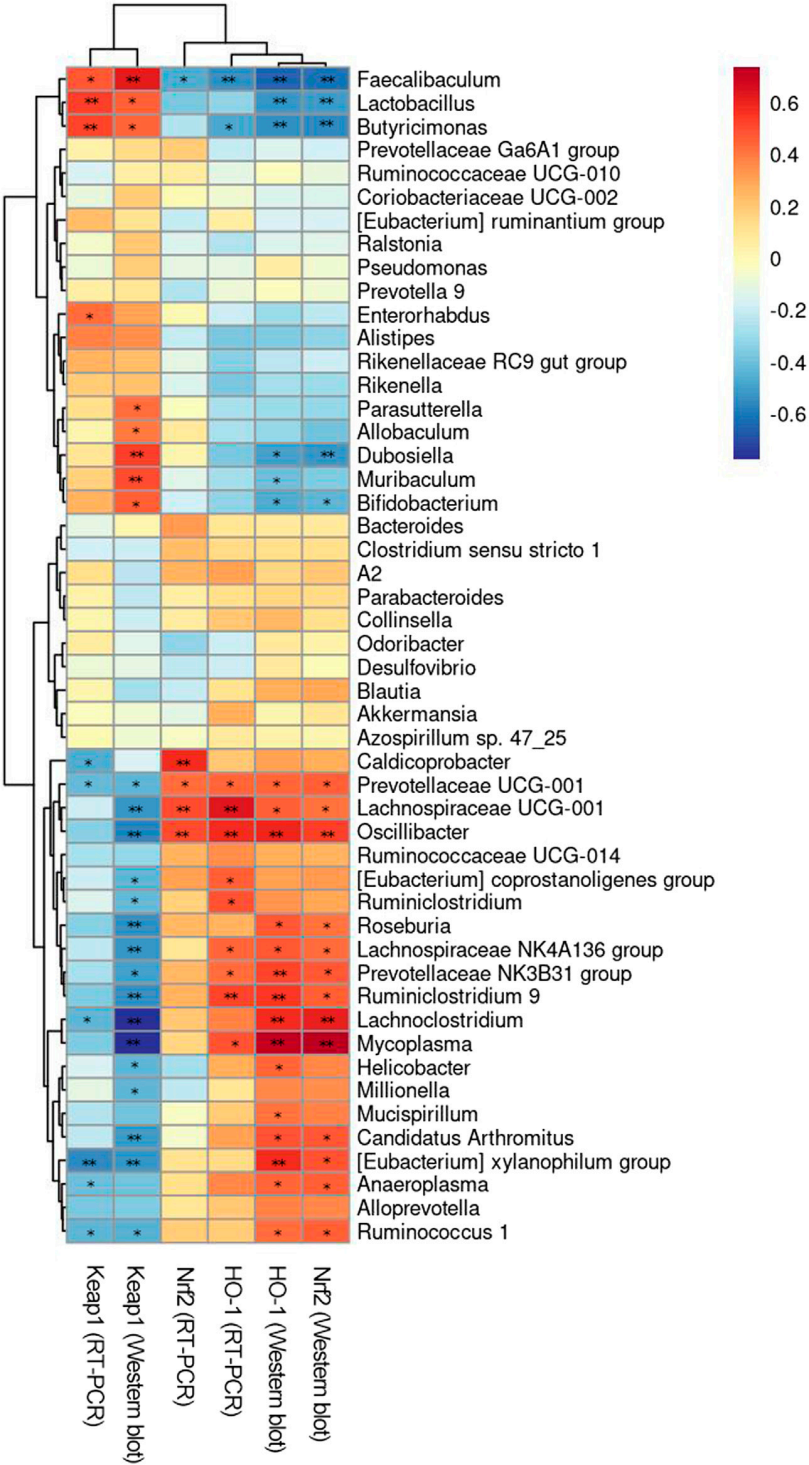
Correlation analysis between intestinal flora and environmental factors. ^*^ *p* < 0.05, ^**^ *p* < 0.01.

As illustrated in Figure 7A, the gut microbiota included 10 phyla, in which *Bacteroidetes*, *Firmicutes*, *Proteobacteria*, *Epsilonbacteraeota*, and *Verrucomicrobia* were dominant in the gut microbiota of each group, and their proportions were 97.88% (CG), 98.61% (MG), 98.43% (PG), 98.90% (RPSG), and 98.26% (WPSG), respectively. Compared with the CG, the proportions of *Proteobacteria* and *Epsilonbacteraeota* decreased, whereas the proportions of *Bacteroidetes*, *Firmicutes*, and *Verrucomicrobia* increased in the MG. After PS treatment, the proportions of *Bacteroidetes* and *Proteobacteria* returned to normal levels. At the genus level, the number detected was 129. As shown in Figure 8B, *Bacteroides*, *Alloprevotella*, *Alistipes*, *Dubosiella,* and *Rikenellaceae RC9 gut group* were dominant in each group. Compared with the CG, the proportions of *Bacteroides* and *Alloprevotella* were lower in the MG. Conversely, the proportions of *Rikenellaceae RC9 gut group, Alistipes*, and *Dubosiella* were upregulated in the MG. After PS treatment, the proportions of *Rikenellaceae RC9 gut group, Bacteroides*, *Alloprevotella*, and *Alistipes* returned to normal levels. The above results indicated that the proportions of the intestinal flora of aging mice was disrupted. After administration, RPS and WPS could regulate the relative abundances of intestinal flora in aging mice to a normal level.

#### 3.7.4 Correction analysis between intestinal flora and environmental factors

Spearman’s rank correlation analysis between the Keap1//Nrf2/ARE signal pathway and intestinal flora of mice was performed to explore the relationship between intestinal flora and environmental factors. As illustrated in Figure 8, the results revealed a notable correlation between the distribution of the intestinal flora in aging mice and the levels of Keap1//Nrf2/ARE in the brain. At the genus level, [*Eubacterium*] *coprostanoligenes group*, [*Eubacterium*] *xylanophilum group*, *Alloprevotella*, *Anaeroplasma*, *Caldicoprobacter*, *Clostridium sensu stricto 1*, *Lachnoclostridium*, *Lachnospiraceae NK4A136 group*, *Lachnospiraceae UCG-001*, *Mycoplasma*, *Oscillibacter*, *Prevotellaceae NK3B31 group*, *Prevotellaceae UCG-001*, *Roseburia*, *Ruminiclostridium*, *Ruminiclostridium 9*, *Ruminococcaceae UCG-014*, and *Ruminococcus one* were positively correlated with the expressions of Nrf2 and HO-1 and negatively correlated with the expression of Keap1. However, *Alistipes*, *Bifidobacterium*, *Butyricimonas*, *Faecalibaculum*, *Lactobacillus*, *Muribaculum*, *Parasutterella*, *Prevotella 9*, *Rikenella*, and *Rikenellaceae RC9 gut group* were negatively correlated with the expression of Nrf2 and HO-1 but positively correlated with the expression of Keap1. [*Eubacterium*] *xylanophilum group*, *Butyricimonas*, *Faecalibaculum*, *Lachnoclostridium*, *Lactobacillus*, *Prevotellaceae UCG-001*, and *Ruminococcus 1* were significantly correlated with the expression of Keap1 at the protein and mRNA levels (*p* < 0.01 or *p* < 0.05). *Faecalibaculum*, *Lachnospiraceae UCG-001*, *Oscillibacter*, and *Prevotellaceae UCG-001* were significantly correlated with the expression of Nrf2 at the protein and mRNA levels (*p* < 0.01 or *p* < 0.05). *Butyricimonas*, *Faecalibaculum*, *Lachnospiraceae NK4A136*, *Lachnospiraceae UCG-001*, *Mycoplasma*, *Oscillibacter*, *Prevotellaceae NK3B31 group*, *Prevotellaceae UCG-001*, and *Ruminiclostridium 9* were significantly correlated with the expression of HO-1 at the protein and mRNA levels (*p* < 0.01 or *p* < 0.05). In conclusion, *Faecalibaculum* and *Prevotellaceae UCG-001* were significantly associated with the activation of Keap1//Nrf2/ARE signal pathway (*p* < 0.01 or *p* < 0.05).

## 4 Discussion

Wine steaming is a unique and effective processing method that is used in the clinical practice of TCM in China. PS is usually processed by steaming to reduce toxicity and promote efficacy before application in a clinic (Shen et al., 2022). The biggest characteristic of TCM is its complex chemical composition, with its multiple components, effects, and targets resulting in its drug effects. UPLC-Q-Orbitrap HRMS has been widely used in the qualitative analysis of TCM because it provides a platform to observe fragment ions of compounds and has the advantages of high sensitivity, high-resolution, high accuracy, and a wide scanning range. The technology not only helps to solve the problem of the diversity and complexity of TCM, but also establishes a foundation for elucidating the therapeutic mechanism of TCM and the development of new drugs (Wong et al., 2016; Liu et al., 2021). In this study, six compounds were detected only in RPS, and 10 compounds were detected only in WPS. Among them, 5-hydroxymethyl-2-furaldehyde and 2-furoic acid were produced by the “Maillard reaction,” a reaction between carbonyl compounds and amino compounds, and the products have been demonstrated to have an effect on antioxidants (Wolfrom et al., 1948; Liu et al., 2014). Saccharides, flavonoids, and alkaloids were the main components that were different in PS before and after processing. The content of D-(−) fructose increased but sucrose decreased after processing, which may be related to the hydrolysis of polysaccharides and oligosaccharides during the process (Chen et al., 2022). Flavonoids, including luteolin, tangeritin, sakuranetin, and nobiletin, were abundant in PS and have proved its therapeutic biological activity. The response intensity of flavonoids differed greatly, and the intensity of amino acids and maltol showed an upward trend after processing, indicating that these components might have transformed into each other during processing (Ren, et al., 2021). The above results demonstrate that the chemical components of RPS and WPS were different and had been transformed, approximately influencing the curative effect of the drugs.

Aging is an irreversible physiological process of functional decline that is mainly manifested by physiological degeneration of cell function, bodily organs, and metabolic functions as well as disordering of gut microbiota. Many studies have shown that the structure of gut microbiota, metabolites, and molecular signals generated during metabolism are closely related to the occurrence of aging diseases (You, 2022). Although PS reportedly has various beneficial effects toward the tonic aspect, there have been few reported studies regarding the mechanism underlying anti-aging effects, especially for modulating gut microbiota. Hence, the mitigating effects of PS on aging were measured by evaluating the capacity of antioxidants, regulating gut microbiota, and the Keap1/Nrf2/ARE signaling pathway.

In this study, the anti-aging effects of RPS and WPS were assessed by establishing a D-gal-induced aging mouse model, which has been widely used in aging studies. Numerous studies have shown that symptoms similar to those of natural aging are induced by long-term intake of high doses of D-gal. Zhao et al. (2017) believed that intraperitoneal injection of D-gal (100–300 mg/kg/day) required continuous modeling for 4–7 weeks. Combined with the study by Chen et al. (2016), the optimal concentration of D-gal to induce a subacute aging mouse model was 500 mg/kg/day. Therefore, 500 mg/kg was chosen as the model-drug dose in this experiment. The mechanism underlying induction of oxidative stress damage is stimulation of excessive oxygen free radicals by D-gal (Han et al., 2014). One study showed that long-term intraperitoneal injection of D-gal induced a variety of biochemical changes in the body, particularly organ degeneration, as observed in the brain and liver (Sheng et al., 2022). Oxidative stress damage theory is the mainstream theory of aging research. Several studies have demonstrated that antioxidant enzymes have a crucial part in removing and decomposing excessive free radicals in the body (Zhang et al., 2021). SOD is one of the important antioxidant enzymes that maintain the body’s antioxidant homeostasis. GSH-PX can prevent peroxides from damaging the body by blocking redox reactions. As a lipid peroxide of cell membranes, MDA can reflect the degree of lipid peroxidation and severity of free-radical attacks in the body (Zhang et al., 2020).

First, compared with the CG, the MG showed worse mental state, lower body weight and spleen index, higher liver index, decreased SOD and GSH-PX activities, and increased MDA levels in the serum and brain tissue, indicating that the aging mouse model was successfully established. Next, the difference in anti-aging efficacy between RPS and WPS was explored. In this study, the effects of RPS and WPS on body weight, pathological effects on the brain, liver index, spleen index, SOD, and MDA, and GSH-PX activity levels of aging mice were recorded. The results showed that both RPS and WPS could significantly improve the mental state, increase body weight, improve the liver and spleen indexes, and reverse the level of antioxidant enzymes in aging mice. However, there were some differences between the effects of RPS and WPS. The overall effect was slightly stronger for WPS than for RPS. In particular, the regulatory effects were significantly better for WPS than for RPS, as shown by increasing the body weight and decreasing liver indexes and promoting serum SOD, brain SOD, and GSH-PX levels in aging mice.

To further clarify these mechanisms, the Keap1/Nrf2/ARE signaling pathway was explored. This pathway is often considered to be linear and primarily mediated by negative feedback beginning with oxidative stress and ending with induction of antioxidant genes (Liu et al., 2022). A previous study showed that loss of Nrf2 promoted aging (Schmidlin et al., 2019). Under normal conditions, Nrf2 is bound and protein degraded by Keap1. When ROS accumulation *in vivo* exceeds the body-scavenging capacity, Keap1 loses its ability to promote Nrf2 ubiquitination and degradation. Then, promotion of the oxidation of Keap1 cysteine residues, induction of the release and activation of Nrf2, mediation of the translocation of Nrf2 to the nucleus, binding of antioxidant response elements, and initiation of the expression of various antioxidant enzymes, such as HO-1 and NQO1 (Xu, 2021), are activated.

At the mRNA and protein levels, the expression of Keap1 strikingly increased in the MG but the expressions of Nrf2 and HO-1 notably decreased compared with those in the MG (*p* < 0.01 or *p* < 0.05). These results indicated that D-gal-induced oxidative stress in the brain tissue of aging mice, especially within the Keap1/Nrf2/ARE signaling pathway, was significantly inhibited. After RPS and WPS intervention, the mRNA levels of Keap1 were significantly decreased, whereas the levels of Nrf2 and HO-1 were significantly increased in the two groups. The protein expression showed the same trend as the mRNA results, and WPS had a stronger normal level restorative effect. We speculated that antioxidant channels might be inhibited in the brain tissue of D-gal-induced aging mice. After administration of RPS and WPS, the anti-oxidative stress Keap1/Nrf2/ARE pathway might be activated to alleviate the symptoms of aging, and the effect of WPS was stronger than RPS.

This study found that intestinal microbiota sharply changed during the model aging process, mainly manifested as reduced abundance and structure, decreased dominant bacteria and proportion of probiotics, and intestinal microecological disorder. The results were consistent with previous research (Wu et al., 2018; Li et al., 2019). In our study, according to the alpha diversity, the Shannon index was decreased, and the Chao1 and PD indexes of fecal microbiota were significantly lower in the MG than in the CG. Compared with the MG, the RPSG showed an increasing trend, whereas the WPSG had an obviously regulatory effect on diversity and richness, indicating that D-gal stimulated specific alterations in the richness and evenness of intestinal microbiota in aging mice. Intervention with RPS and WPS can improve the diversity of gut microbiota, and the effect is better with WPS than with RPS. Based on the Beta diversity, in the MG and the CG, the gut microbiota structure was notably changed showing that RPS and WPS treatment could restore normal levels.

Numerous studies have demonstrated that neurodegenerative disease-related oxidative stress, such as that caused by aging, Alzheimer’s disease, and Parkinson’s disease, show a reduced relative abundance of *Firmicutes* (Arboleya et al., 2016; Xiong et al., 2021; Hang et al., 2022). *Verrucomicrobia* has beneficial effects of prolonging health and life spans (Jandhyala et al., 2015). Zhao et al. (2018) found that compared with normal mice, aging mice had an increased proportion of F/B (*Firmicutes*/*Bactroidetes*) in the intestinal microbiota, and the variety of beneficial bacteria was decreased. At the phylum level, after administration of RPS and WPS, the relative abundance of *Firmicutes* and the ratio of F/B increased relative to those in the MG, and the relative abundance of *Verrucomicrobia* also increased. WPS outperformed RPS in regulation of *Firmicutes* and *Bacteroidetes*. At the genus level, compared with the CG, the MG showed decreased relative abundances of *Bacteroides*, *Alloprevotella*, and *Helicobacter*. After administration of RPS and WPS, the relative abundances of *Bacteroides*, *Alloprevotella*, *Dubosiella,* and *Akkermansia* were increased, whereas the relative abundances of the *Rikenellaceae RC9 gut group*, *Butyricimonas*, and *Parasutterella* were decreased. A previous study showed that along with increasing age, the cumulative abundances of *Alloprevotella*, *Dubosiella*, and *Akkermansia* associated with healthy groups were decreased (Biagi et al., 2016). *Bacteroides* is considered to be a beneficial bacteria that produces abundant SCFAs, which participate in mediating the oxidation and inflammation levels in the brain and gut that effectively enhances immunity and postpones aging (Hao et al., 2022).

D-gal-induced aging has been shown to be related to changes in the gut microbiota structure. The expressions of Keap1, Nrf2, and HO-1 in brain tissue are also closely related to aging. In the brain–gut axis, a functional relationship that mediates between the gut microbiota and brain behavior has been previously observed (Ling et al., 2022). Therefore, we suspected that the expressions of Keap1, Nrf2, and HO-1 in brain tissue may be related to the intestinal flora. Therefore, we performed Spearman’s rank correlation analysis. The correlation results and changes in the structure of the intestinal flora at the genus level showed that the expressions of Nrf2, HO-1, and Keap1 in the brain of aging mice were correlated with the relative abundances of the [*Eubacterium*] *coprostanoligenes group*, [*Eubacterium*] *xylanophilum group*, *Alloprevotella*, *Anaeroplasma*, *Caldicoprobacter*, *Clostridium sensu stricto 1*, *Lachnoclostridium*, *Lachnospiraceae NK4A136 group*, *Lachnospiraceae UCG-001*, *Mycoplasma*, *Oscillibacter*, *Prevotellaceae NK3B31 group*, *Prevotellaceae UCG-001*, *Roseburia*, *Ruminiclostridium*, *Ruminiclostridium 9*, *Ruminococcaceae UCG-014*, *Ruminococcus 1, Alistipes*, *Bifidobacterium*, *Butyricimonas*, *Faecalibaculum*, *Lactobacillus*, *Muribaculum*, *Parasutterella*, *Prevotella 9*, *Rikenellaceae RC9 gut group*, *Rikenella,* and (*p* < 0.01 or *p* < 0.05). The RT-PCR and WB results indicate that the protein and mRNA expressions of Keap1 in PS might be related to the abundances of the [*Eubacterium*] *xylanophilum group*, *Butyricimonas*, *Faecalibaculum*, *Lachnoclostridium*, *Lactobacillus*, *Prevotellaceae UCG-001*, and *Ruminococcus 1* (*p* < 0.01 or *p* < 0.05). The protein and mRNA expressions of Nrf2 in PS might be related to *Faecalibaculum*, *Lachnospiraceae UCG-001*, *Oscillibacter*, and *Prevotellaceae UCG-001* (*p* < 0.01 or *p* < 0.05)*.* The protein and mRNA expressions of HO-1 in PS might be related to *Butyricimonas*, *Faecalibaculum*, *Lachnospiraceae NK4A136 group*, *Lachnospiraceae UCG-001*, *Mycoplasma*, *Oscillibacter*, *Prevotellaceae NK3B31 group*, *Prevotellaceae UCG-001*, and *Ruminiclostridium 9* (*p* < 0.01 or *p* < 0.05). Finally, *Faecalibaculum* and *Prevotellaceae UCG-001* were significantly related to the expressions of the components in the Keap1//Nrf2/ARE signal pathway (*p* < 0.01 or *p* < 0.05).

This study had some limitations. The study results were obtained from elementary data that showed treatment with RPS and WPS could notably improve the antioxidant capacity in serum and the brain and modulate the gut microbiota. However, future studies should focus on the transformation of gut microbiota.

## 5 Conclusion

Different intensities of pharmacological effects can be observed between RPS and WPS. The study confirmed that RPS and WPS had a positive effect on mediating D-gal-induced aging. WPS treatment outperformed RPS in reversing the D-gal-induced changes, including increasing the spleen index and SOD and GSH-PX activities and decreasing the liver index and MDA levels. Furthermore, the therapeutic mechanism of RPS and WPS was related to alleviating oxidative stress by activating the Keap1/Nrf2/ARE signal pathway and regulating the structure of gut microbiota. This study provides a comprehensive reference for the effect of processed PS products on aging pathogenesis and provides a basis for the rational clinical use of processed products.

## Data Availability

The datasets presented in this study are deposited in the SRA repository, accession number PRJNA1089844, available at https://www.ncbi.nlm.nih.gov/sra/PRJNA1089844.

## Abbreviations

ARE, Antioxidant response elements; D-gal, D-galactose; GSH-PX, Glutathione peroxidase; HE, Hematoxylin and eosin; HO-1, Heme oxygenase 1; Keap1,.Kelch-like ECH-associated protein 1; MDA, Malondialdehyde; Nrf2, Nuclear factor erythroid 2-related factor 2; PS, Polygonatum sibiricum; RPS, Raw Polygonatum sibiricum; ROS, Reactive oxygen species; RT-PCR, Reverse transcription-polymerase chain reaction; SCFAs, Short-chain fatty acids; SOD, Superoxide dismutase; TCM, Traditional Chinese medicine; UPLC-Q-Orbitrap HRMS, Ultra-performance liquid chromatography–hybrid quadrupole-Orbitrap high-resolution mass spectrometry; WB, Western blot; WPS, Wine-steamed Polygonatum sibiricum; 16S rRNA, 16S ribosomal RNA.
